# Adiposity, inflammation, and working memory: Evidence for a vicious cycle

**DOI:** 10.1016/j.bbih.2021.100202

**Published:** 2021-01-09

**Authors:** Grant S. Shields, LillyBelle K. Deer, Paul D. Hastings, Camelia E. Hostinar

**Affiliations:** a Center for Mind and Brain, University of California, Davis, CA, United States; b Department of Psychology, University of California, Davis, CA, United States

**Keywords:** Working memory, Inflammation, Adiposity, Overweight, Obesity, CRP, ALSPAC

## Abstract

Overweight and obesity constitute the fifth leading cause of preventable deaths worldwide. One pathway through which excess weight contributes to poor health outcomes is via inflammatory activity and changes in cognitive processes. Prior theory has proposed a vicious cycle whereby obesity potentiates inflammatory activity, which alters cognitive processes such as working memory, which in turn leads to a reduced ability to self-regulate and therefore manage weight. However, to date no longitudinal studies have examined this potential dynamic. In the current study, we addressed this gap by assessing the relations among fat mass, C-reactive protein (CRP), and working memory across time in a large sample of 8536 children followed through adolescence in the Avon Longitudinal Study of Parents and Children in the United Kingdom. Adiposity was quantified via dual emission x-ray absorptiometry (DEXA) at ages 9 and 15.5 years old, and inflammatory activity was indexed via circulating serum C-reactive protein (CRP) levels assessed with a high-sensitivity assay at those same ages. Working memory was assessed between these two time points, at age 10, permitting examination of the temporal relations between working memory, adiposity, and inflammatory activity. As hypothesized, we found that fat mass predicted later poor working memory, and this association was statistically mediated by CRP. Further, we found that poor working memory predicted greater subsequent fat mass and CRP, and the link between working memory and subsequent CRP was partially mediated by fat mass. These results thus could be taken to suggest the existence of a vicious cycle of mutually amplifying adiposity, inflammatory activity, and poor working memory over time.

## Highlights

• Adiposity and inflammatory activity (CRP) was measured at ages 9 and 15.5 ​yrs.
• Working memory was assessed at age 10 ​yrs.
• Adiposity predicted working memory, and this association was mediated by CRP.
• Working memory predicted later adiposity and CRP, this was mediated by adiposity.

## 1 Introduction

The growing prevalence of overweight and obesity has been called a global public health crisis (Karnik and Kanekar, 2012; Lobstein et al., 2004). In fact, overweight and obesity constitute the fifth leading cause of preventable deaths worldwide, responsible for 4.72 million deaths in 2017 (GBD 2017 Risk Factor Collaborators, 2018). These statistics have prompted a wealth of research aimed at understanding the health consequences and factors that contribute to the development or maintenance of adiposity (e.g., Cazettes et al., 2011; Shields et al., 2017; Slavich, 2015; Verbeken et al., 2013a; Yokum and Stice, 2013). For example, adipose tissue contributes to inflammation (Hotamisligil, 2006), which predisposes to numerous chronic diseases, such as coronary heart disease and depression (Libby et al., 2018; Miller and Raison, 2016). Some recent evidence also highlights cognitive sequelae of elevated inflammation, such as impairments in executive function skills, which may contribute to the accumulation of excessive weight (Dohle et al., 2018; Yang et al, 2018, 2019a), fueling a vicious cycle of mutually reinforcing adiposity, inflammatory activity, and executive function deficits over time (Shields et al., 2017). One specific executive function skill that has been posited as important is working memory (Dohle et al., 2018), which refers to the ability to keep information in mind and update one’s mental contents when appropriate. Excess weight and adiposity have been linked to poorer working memory (Yang et al, 2018, 2020), and links between adiposity and working memory have been proposed to be related to or mediated by inflammatory activity (Yang et al., 2020). However, to date no study has examined longitudinal relations between working memory, inflammatory activity, and adiposity, to provide support for the existence of a potential vicious cycle.

There are at least two potential pathways through which adiposity would be linked to working memory. The first of these potential pathways is that adiposity may impair working memory via inflammatory activity (Shields et al., 2017; Yang et al., 2020). In support of this, inflammatory activity produces functional and, over time, structural changes in the brain through stimulation of the vagus nerve, activation of microglia, and direct effects on neurons (Dantzer and Kelley, 2007; de Pablos et al., 2006; Harrison et al., 2015; Shields et al., 2017; Zalcman et al., 1994). Notably, inflammatory activity alters neural activity within the dorsolateral prefrontal cortex (Harrison et al., 2009), and the dorsolateral prefrontal cortex is a key brain region underpinning working memory performance (Bogdanov and Schwabe, 2016). More directly, nonhuman animal work has shown that inflammatory activity impairs working memory in rodents (Chen et al., 2008; Sparkman et al., 2006). Because adipocytes produce proinflammatory cytokines (Coppack, 2001), these findings have led some to theorize that a primary pathway linking adiposity to poor working memory is inflammatory activity (Shields et al., 2017; Yang et al., 2020).

The second potential pathway linking adiposity and working memory contends that there may be a direct beneficial effect of working memory on health-related eating behaviors and thus weight (Dohle et al., 2018; Hostinar et al., 2015; Yang et al., 2018). In particular, working memory has been proposed as a key component of self-regulation (Hofmann et al., 2012), primarily through enabling the mental representation and maintenance of long-term goals (Dohle et al., 2018). In particular, working memory is critical for keeping goals in mind and thus maintaining goal-directed behavior when faced with temptations or distractions (Dohle et al., 2018; Hofmann et al., 2012, 2013). Additionally, working memory also supports other components of self-regulation. For example, by enabling representation of multiple interpretations of an event, working memory capacity facilitates cognitive reappraisal in emotion regulation (Ilkowska and Engle, 2010; Schmeichel et al., 2008). Similarly, working memory can support inhibitory control (i.e., the ability to inhibit thoughts or prepotent responses in order to engage in goal-directed rather than habitual actions; Diamond, 2013) because maintaining a mental representation of goals can make it easier to refrain from engaging in habitual actions (Dohle et al., 2018; Hofmann et al., 2012, 2013). Within the context of eating behavior and adiposity, better working memory is thought to facilitate mental maintenance of health- and diet-related goals and therefore reduce desire for unhealthy, goal-opposing food (Allom and Mullan, 2014; Dassen et al., 2018; Dohle et al., 2018; Goldschmidt et al., 2018; Hofmann et al., 2012, 2013; Whitelock et al., 2018). In support of the idea that working memory influences weight and adiposity, working memory training interventions have been found to improve eating behavior and decrease weight (Houben et al., 2016; Verbeken et al., 2013a). In this pathway, then, working memory impacts inflammatory activity through effects on body weight.

These two pathways are not mutually exclusive. For example, it is certainly possible that these factors set in motion a vicious cycle over time. In this framework, adiposity leads to inflammation, which leads to poorer working memory. Then, through reduced ability to keep long-term food-related goals in mind—and therefore leading to detrimental health behaviors such as poor diet—poor working memory is thereby thought to lead to greater adiposity and inflammatory activity, then further impairments in working memory over time.

The idea that poor working memory, adiposity, and inflammatory activity may influence each other over time has particularly important implications for understanding development. For example, adolescence is typically a time of marked changes in working memory (Luciana et al., 2005), body mass (Kimm et al., 2005), and the immune system (Brenhouse and Schwarz, 2016). As a result, this may be a time when the vicious cycle of increasing adiposity, inflammatory activity, and working memory deficits may begin to take hold, with ramifications for lifelong health (Shields et al., 2017). More importantly, adolescence presents a “window of opportunity” for interventions that can influence health and cognitive development as well as for understanding health and cognitive development in general (Dorn et al., 2019). For example, adolescence marks a unique time wherein both working memory and the immune system go through a marked period of maturation contemporaneously (Brenhouse and Schwarz, 2016; Luciana et al., 2005). Indeed, adolescence has been argued to be a sensitive period for both brain and immune system development (Brenhouse and Schwarz, 2016; Schulz et al., 2009). Despite the importance of adolescent adiposity and working memory in predicting lifelong health outcomes (e.g., Reilly and Kelly, 2011; Suchy et al., 2016), however, to date no research has examined how working memory, adiposity, and inflammatory activity relate to each other over time during adolescence.

### 1.1 Current research

The current study aims to address gaps in the literature by assessing the longitudinal relations between working memory, adiposity, and inflammatory activity in a sample of 8536 children followed through adolescence from the Avon Longitudinal Study of Parents and Children in the United Kingdom. Although many cognitive processes are related to both adiposity and inflammatory activity—for example, long-term memory (Harrison et al., 2014; Loprinzi and Frith, 2018; Marsland et al., 2008) or cognitive inhibition (Shields et al., 2020; Yang et al., 2018, 2019a)—we chose to focus on working memory rather than other cognitive processes because we recently found cross-sectional evidence for a statistical mediation of an association between body mass index and working memory by C-reactive protein (CRP; Yang et al., 2020). However, the entirely cross-sectional nature of that study and the imprecise method used to quantify adiposity limited inferences that could be made. Moreover, this study did not examine these dynamics during an age in which working memory is highly malleable (e.g., adolescence; Diamond, 2013; Peeters et al., 2014). To address these issues, we examined longitudinal relations between working memory and both adiposity and CRP measured with a high-sensitivity assay (hs-CRP) in a sample of adolescents in the present study.

Adiposity was quantified via dual emission x-ray absorptiometry (DEXA) at two time points relevant to this study—ages 9 and 15.5 years old. Inflammatory activity was indexed via circulating CRP levels assayed from serum at the same ages, 9 and 15.5. Working memory was assessed between these two time points, at age 10, thus permitting examination of the temporal relations between working memory, adiposity, and inflammatory activity. We hypothesized a reciprocal relation between these variables over time. Namely, drawing on the first potential pathway described, we hypothesized that CRP would statistically mediate the association between adiposity and subsequent working memory; similarly, drawing on the second potential pathway described, we hypothesized that adiposity would statistically mediate the association between working memory and subsequent CRP.

## 2 Method

### 2.1 Participants

Data in these analyses were from the Avon Longitudinal Study of Parents and Children (ALSPAC). ALSPAC is an ongoing birth cohort study that aims to follow participants from birth into adulthood to understand the role of environmental and genetic factors in shaping a wide range of developmental and health outcomes. Mothers were recruited if they had an expected delivery date between April 1, 1991 and December 31, 1992 and lived in the former county of Avon in the United Kingdom. This recruitment procedure resulted in an initial sample of 14,541 pregnant mothers. When the oldest children were approximately 7 years of age, an attempt was made to bolster the initial sample with eligible cases who had failed to join the study originally, resulting in a total sample size of 15,454 pregnancies, with 14,901 of those children being alive at age one. For further information regarding sample enrollment, participant characteristics, and general study methodology, we refer the reader to publications from the ALSPAC team that have profiled this cohort (Boyd et al., 2013; Fraser et al., 2013; Golding et al., 2001). Please note that the study website contains details of all the data that are available through a fully searchable data dictionary and variable search tool (http://www.bristol.ac.uk/alspac/researchers/our-data/). There were 8536 youth who had hs-CRP or fat mass data available at ages 9 or 15.5 or working memory data (50.0% female, 2.2% declined to state or had missing gender information; 92.9% White/Caucasian, 4.9% declined to state or had missing race/ethnicity information). The ALSPAC study did not have any exclusion criteria for adiposity, CRP, or working memory measurement. Although we report analyses excluding participants with CRP values greater than 10 ​mg/L (see below), we chose to retain all participants in analyses by default in order to enhance the generalizability of our results.

### 2.2 Materials

#### 2.2.1 Fat mass

Fat mass was measured at ages 9 and 15.5 years old using a Lunar Prodigy dual emission x-ray absorptiometry (DEXA) scanner (GE Medical Systems Lunar). DEXA is considered the gold standard for measurement of fat mass and is more precise than other methods for measuring adiposity (Gupta et al., 2011; Riddoch et al., 2009). All scans were manually examined for motion, other artifacts, and anomalies. Total mass estimated by the DEXA scan was virtually identical to measured mass in this study, *R*^2^>0.99 (Riddoch et al., 2009). Fat mass (in grams) was available and used as the quantification of adiposity at age 9, and fat mass (in grams) was available and used as the quantification of adiposity at age 15.5.

#### 2.2.2 C-reactive protein

Participants provided blood samples during clinical assessments at ages 9 and 15.5. Participants fasted overnight for morning assessments or for 6 ​h prior for afternoon assessments. Blood samples were immediately spun and then frozen at −80 ​°C. Samples were stored for up to 9 months without any intervening freeze-thaw cycles. hs-CRP was quantified from these samples using a high-sensitivity automated particle-enhanced immunoturbidimetric assay (Roche UK, Welwyn Garden City, UK). All interassay coefficients of variation were less than 5%.

#### 2.2.3 Working memory

Working memory at age 10 was assessed using a computerized version of the Counting Span Task (Case et al., 1982). On each trial, participants viewed a screen consisting of red and blue dots against a white background, and participants were asked to both point to and count the number of red dots aloud. The task began with two practice sets of two trials each. After completing the practice sets, participants then completed three sets of two trials each, three sets of three trials each, three sets of four trials each, and three sets of five trials each. After each set, participants were required to report the number of red dots they had seen on each trial in the order that each trial was presented during the set. All participants completed all sets regardless of their performance. Working memory was quantified in this study as the number of trials a participant answered correctly (i.e., the “global” score in ALSPAC) due to prior work establishing that these partial credit scores have better reliability than more traditional all-or-nothing span scores (Conway et al., 2005). However, results were equivalent when the ALSPAC “span” score was used instead of the “global” score—all significant paths remained significant, and no non-significant paths became significant.

### 2.3 Procedure

Participants were assessed at three different time points relevant to this study: ages 9, 10, and 15.5 years old. Participants completed clinical assessments at ages 9 and 15.5, when fat mass was measured using DEXA and blood was drawn for quantification of hs-CRP. At age 10, in contrast, participants completed a number of questionnaires and other tasks, including, most notably for purposes of this study, the working memory task. Participants also completed assessments at other time points, from which relevant covariates were drawn if they were not assessed contemporaneously with the primary study variables (e.g., family income at child age 11). Ethical approval for the study was obtained from the ALSPAC Ethics and Law Committee and the Local Research Ethics Committees. Informed consent for the use of the data collected via questionnaires and clinics was obtained from participants following the recommendations of the ALSPAC Ethics and Law Committee at the time. Consent for biological samples has been collected in accordance with the Human Tissue Act (2004).

### 2.4 Data analysis

All variables were examined for skewness and log transformed when there was evidence of substantial skew (i.e., skewness statistic ​> ​1); fat mass at ages 9 and 15.5 as well as hs-CRP at ages 9 and 15.5 all evidenced substantial positive skew and were therefore log transformed to meet statistical assumptions of normality. Bivariate associations between variables were assessed using standard Pearson correlation tests. Our primary analyses were structural equation models conducted using lavaan, version 0.6–6, in R, version 4.0.0. Missing data in these structural equation models were estimated using full information maximum likelihood. It should be noted that missingness was nonrandom in these data, χ^2^(75) ​= ​135.1, *p* ​< ​.001.^1^ In analyses including covariates, we covaried self-reported gender, race/ethnicity, family income at child age 11, pubertal status at ages 9 and 15.5, and hospitalizations between the ages of 9 and 13 given known effects or associations of gender, race/ethnicity, socioeconomic status, pubertal status, and health with CRP, adiposity, or working memory (Gold et al., 2010; O’Connor et al., 2009; Yang et al., 2019b). Adiposity and hs-CRP were autoregressed on their earlier timepoints to control for prior values of these variables in analyses.

## 3 Results

Descriptive statistics for the primary variables of interest are presented in Table 1.

**Table 1:** Descriptive statistics and correlations for primary variables of interest.

Variable	*M*	*SD*	1	2	3	4
1. Working memory	18.52	(7.62)				
*n* ​= ​7006						
2. hs-CRP, age 9 (mg/L, ln)	−1.30	(1.22)	-.05∗∗∗			
*n* ​= ​5082			[-.08, −.02]			
3. hs-CRP, age 15.5 (mg/L, ln)	−.69	(1.11)	-.08∗∗∗	.30∗∗∗		
*n* ​= ​3488			[-.11, −.04]	[.26, .33]		
4. Fat mass, age 9 (g, ln)	8.89	(.58)	-.04∗∗	.43∗∗∗	.25∗∗∗	
*n* ​= ​7329			[-.06, −.01]	[.41, .45]	[.22, .29]	
5. Fat mass, age 15.5 (g, ln)	9.46	(.61)	-.06∗∗∗	.35∗∗∗	.27∗∗∗	.77∗∗∗
*n* ​= ​5147			[-.09, −.03]	[.32, .38]	[.24, .31]	[.76, .78]

*Note*: ∗∗*p* ​< ​.01, ∗∗∗*p* ​< ​.001; *M* ​= ​mean; *SD* ​= ​standard deviation; hs-CRP ​= ​high sensitivity C-reactive protein; mg/L ​= ​milligrams per liter; g ​= ​grams; ln ​= ​log natural transformed. Fat mass was measured by dual emission x-ray absorptiometry (DEXA). Values in brackets represent 95% confidence intervals of each Pearson correlation coefficient.

To examine links necessary for any potential mediational pathways between adiposity, CRP, and working memory, we first assessed bivariate associations among these variables, starting with whether adiposity related to CRP levels. As expected, greater adiposity was significantly associated with higher CRP levels at both age 9, *r*(4838) ​= ​0.431, *p* ​< ​.001, and age 15.5, *r*(3265) ​= ​0.274, *p* ​< ​.001. Next, we examined whether working memory at age 10 was associated with adiposity at either age. As expected, greater adiposity at age 9 was a significant predictor of worse working memory at age 10, *r*(6163) ​= ​−0.038, *p* ​= ​.003, and worse working memory at age 10 was a significant predictor of greater adiposity at age 15.5, *r*(4445) ​= ​−0.056, *p* ​< ​.001—suggestive of a small but significant bidirectional relationship between these variables that unfolds over time. In our final set of bivariate analyses, we found that, as expected, higher CRP at age 9 was a significant predictor of worse working memory at age 10, *r*(4287) ​= ​−0.054, *p* ​< ​.001, and worse working memory at age 10 was a significant predictor of higher CRP levels at age 15.5, *r*(2922) ​= ​−0.078, *p* ​< ​.001.

### 3.1 Primary analyses

**Fig. 1:**
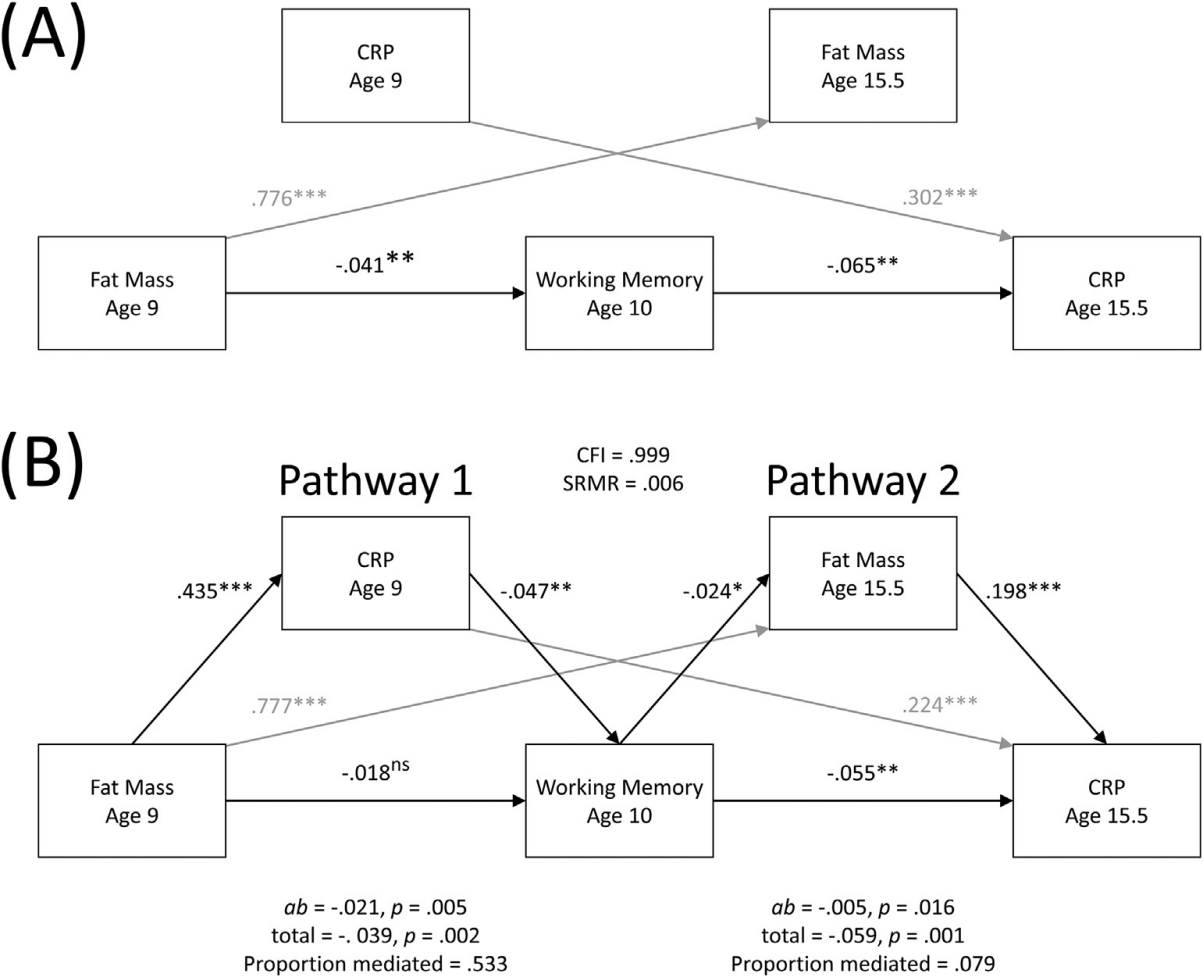
Associations among fat mass, CRP, and working memory over time without theorized mediational pathways (A), and mediation model testing indirect associations in links between fat mass, CRP, and working memory over time (B). In examining Pathway 1, the link between adiposity at age 9 and working memory at age 10 was statistically mediated by CRP at age 9, as hypothesized. Similarly, in Pathway 2, the link between working memory at age 10 and CRP at age 15.5 was statistically mediated by adiposity at age 15.5, again as hypothesized. When adjusting for covariates (see main text), both mediations remained significant. Autoregressive paths are grayed out for ease of viewing the primary paths of interest. All coefficients are standardized (β) coefficients.

To test the two potential pathways described above (i.e., Pathway 1: adiposity predicts subsequent working memory through inflammatory activity; Pathway 2: working memory predicts subsequent inflammatory activity through adiposity), we fit the data to a dual mediation structural equation model, which is depicted in Fig. 1B. This model had a good fit to the data, χ^2^(2) ​= ​5.85, *p* ​= ​.054, CFI ​= ​0.999, TLI ​= ​0.997, RMSEA ​= ​0.015, SRMR ​= ​0.006, BIC ​= ​74210.22.^2^

As hypothesized, adiposity at age 9 was significantly associated with CRP at age 9, β ​= ​0.435, *p* ​< ​.001, and CRP at age 9 was a significant predictor of worse working memory at age 10, β ​= ​−0.047, *p* ​= ​.005. Critically, consistent with Pathway 1, CRP at age 9 statistically mediated the link between adiposity at age 9 and working memory at age 10: indirect pathway β ​= ​−0.021, *p* ​= ​.005. Moreover, although the total association between age 9 adiposity and age 10 working memory was significant, β ​= ​−0.041, *p* ​= ​.002 (Fig. 1A), this association became nonsignificant (β ​= ​−0.018, *p* ​= ​.216) when accounting for the proportion of that association explained by CRP (proportion mediated ​= ​.533). These results therefore indicate that adiposity may relate to poorer working memory over time in part through CRP levels.

Next, we tested the predictions described by Pathway 2. As hypothesized, worse working memory at age 10 was a significant predictor of greater adiposity at age 15.5, β ​= ​−0.024, *p* ​= ​.014, and greater adiposity at age 15.5 was significantly associated with higher CRP levels at age 15.5, β ​= ​0.198, *p* ​< ​.001. Critically, consistent with Pathway 2, adiposity at age 15.5 statistically mediated the link between working memory at age 10 and CRP at age 15.5: indirect pathway β ​= ​−0.005, *p* ​= ​.016. In contrast to the above, however, the association between working memory at age 10 and CRP at age 15.5 (total β ​= ​−0.059, *p* ​= ​.001) remained significant (β ​= ​−0.055, *p* ​= ​.002) even after accounting for the proportion of this association mediated by adiposity (proportion mediated ​= ​.079). These findings did not change when CRP values greater than 10 ​mg/L were excluded.^3^ As described above, we also regressed adiposity at age 15.5 on adiposity at age 9 and CRP at age 15.5 on CRP at age 9; we found that, as expected adiposity at age 9 was a significant predictor of adiposity at age 15.5, β ​= ​0.777, *p* ​< ​.001, and CRP at age 9 was a significant predictor of CRP at age 15.5, β ​= ​0.224, *p* ​< ​.001.

Controlling for gender, race/ethnicity, family socioeconomic status, pubertal status at ages 9 and 15.5, and hospitalizations from age 9 to age 13 (model CFI ​= ​0.999, SRMR ​= ​0.006) did not alter the results presented above. In particular, Pathway 1 (i.e., the link between adiposity and future working memory being mediated by CRP) and Pathway 2 (i.e., the link between working memory and future CRP being mediated by adiposity) both remained significant (Pathway 1, mediation by CRP: β ​= ​−0.020, *p* ​= ​.006, proportion mediated ​= ​.514; Pathway 2, mediation by adiposity: β ​= ​−0.005, *p* ​= ​.014, proportion mediated ​= ​.075). In sum, although the strength of indirect association and the proportion mediated was numerically stronger for Pathway 1 (i.e., mediation of the association between adiposity and working memory by CRP) than Pathway 2 (i.e., mediation of the association between working memory and CRP by adiposity), both of these pathways found support in these data and therefore help to elucidate the relations between adiposity, CRP, and working memory over time.

### 3.2 Alternative mediation model analyses

Although the mediation models we tested were theoretically motivated, the contemporaneous assessment of adiposity and CRP entails that an alternative model should be examined. Therefore, we examined a model where adiposity at age 9 mediated the link between CRP at 9 and working memory at age 10, and where CRP at age 15.5 mediated the link between working memory at age 10 and adiposity at age 15.5. This model (BIC ​= ​74277.20; χ^2^(2) ​= ​72.82, *p* ​< ​.001; CFI ​= ​0.987; TLI ​= ​0.936; RMSEA ​= ​0.064, 90% CI_RMSEA_ [0.052, 0.077]; SRMR ​= ​0.036) was a notably worse fit than our theoretically driven model outlined above (BIC ​= ​74210.22; χ^2^(2) ​= ​5.85, *p* ​= ​.054; CFI ​= ​0.999; TLI ​= ​0.997; RMSEA ​= ​0.015, 90% CI_RMSEA_ [0.000, 0.030]; SRMR ​= ​0.006), ΔBIC ​= ​66.98. Therefore, the links between adiposity, CRP, and working memory over time are better explained by our Fig. 1B model than an alternative mediation model.

## 4 Discussion

The prevalence of overweight and obesity has risen tremendously in recent decades, and this continued rise brings with it enormous consequences for public health. It is particularly important to study obesity during childhood and adolescence, due to distinct life trajectories brought about by earlier onset of obesity, as well as the possibility for intervention during this time (Karnik and Kanekar, 2012; Lobstein et al., 2004; Reilly and Kelly, 2011). Prior work has suggested that poor working memory may be both a cause and a consequence of adiposity as well as adiposity-induced inflammatory activity, but to date no study had examined the relations between working memory and both adiposity and inflammatory activity across time. In the present study, we found support for two predicted pathways linking these variables in the large ALSPAC sample of children followed through adolescence and beyond. First, greater adiposity at age 9 predicted poorer working memory at age 10 through greater CRP at age 9. Second, poorer working memory at age 10 predicted greater CRP at age 15.5 through greater adiposity at age 15.5. Notably, these mediational links fit better than an alternative model that linked CRP at age 9 to working memory at age 10 through adiposity at age 9 and then linked poorer working memory at age 10 to adiposity at age 15.5 through CRP at age 15.5. Therefore, although correlational, these results therefore could be taken to suggest that adiposity, inflammatory activity, and working memory may influence each other over time, which has important implications for understanding the development and maintenance of overweight and obesity.

The first potential pathway we examined for relations among adiposity, inflammatory activity, and working memory was that adiposity would predict poorer working memory over time, and this link would be mediated by inflammatory activity. The results supported the existence of this pathway. Our results are therefore consistent with much cross-sectional work linking excess weight to poorer working memory (for a meta-analysis, see Yang et al., 2018), work finding that CRP or hs-CRP is associated with cognitive abnormalities in obesity (Gunathilake et al., 2016; Lasselin et al., 2016; Leigh and Morris, 2020; Levine and Crimmins, 2012; Miller and Spencer, 2014; Spyridaki et al., 2016; Sweat et al., 2008), and a cross-sectional study that found that a marker of inflammatory activity statistically mediated the association between body mass index and working memory in adults (Yang et al., 2020). More broadly, our results are consistent with theoretical work suggesting that inflammatory activity may impair executive functions, including working memory, through its effects on prefrontal cortex structure and function (Hostinar et al., 2018; McAfoose and Baune, 2009; Nusslock and Miller, 2016; Shields et al., 2017).

The second potential pathway we examined for relations among adiposity, inflammatory activity, and working memory was that poorer working memory would predict greater adiposity over time, and through adiposity working memory would indirectly predict greater inflammatory activity over time. Consistent with prior work suggesting that working memory training can improve weight in children (Verbeken et al., 2013b; see also Houben et al., 2016) and that excess weight increases inflammatory activity (Cazettes et al., 2011; Coppack, 2001; Hsuchou et al., 2012), the results supported the existence of this pathway. Within this analysis, however, two notable findings that merit discussion emerged. First, although the indirect pathway between working memory and inflammatory activity through adiposity was significant, the direct pathway between working memory and inflammatory activity remained significant even when accounting for the indirect association. The second notable finding, related to the first, was that the proportion of variance explained in the link between working memory and inflammatory activity by adiposity was much lower than the proportion of variance explained by the indirect association in the first pathway. Together, these findings indicate that working memory predicts subsequent inflammatory activity through more than adiposity alone. We can only speculate about what some of these pathways could be, but one potential candidate is substance use or abuse. For example, poorer working memory is associated with a greater likelihood of smoking, alcohol consumption, and illicit drug use in adolescents (Khurana et al., 2017; Patterson et al., 2010; Peeters et al., 2014), and use or abuse of these substances increases inflammatory activity (O’Connor et al., 2009). These findings therefore support models which posit that regulatory cognitive functions support a variety of health behaviors—including eating-related health behaviors—and these health behaviors link those cognitive functions to subsequent inflammation (Hostinar et al., 2015).

The magnitude of correlations between working memory and adiposity or inflammatory activity were relatively small by standard convention (i.e., the strongest correlation with working memory was *r* ​= ​0.078), which therefore merits discussion. First and foremost, the sizes of the associations we found suggest that there may be a number of state and trait factors linked to adiposity, CRP, and working memory that were not included in our models. Therefore, despite the mediational links we found among these variables, it is important to note that many other factors should be considered when attempting to understand each of these variables. Nonetheless, although these associations were relatively small, the consequences of these associations are far from small. In particular, developmental effects are cumulative, and small differences in important factors can add up over time (Lamm et al., 2014). Working memory and adiposity are each predictive of numerous important lifelong outcomes (Diamond, 2013; Reilly and Kelly, 2011) and small differences in either of these variables could therefore result in vastly different trajectories in life. Moreover, although correlational, our results could be taken as evidence for a vicious cycle, entailing that adiposity may result in greater adiposity through inflammatory activity and worse working memory, further upregulating inflammatory activity and impairing working memory, leading to exponentially greater effects over the lifespan. Therefore, although the magnitude of the bidirectional associations we examined was small, it is possible that the importance of these associations magnifies over time.

This study is not without limitations. First, the data were correlational, which precludes causal inference. The longitudinal design permits inference of temporal precedence in how working memory relates to adiposity and CRP, but cause cannot be determined from our results. Second, as with any longitudinal study, participant attrition occurred between time points, entailing that we were missing data on some measures from participants at different time points. Importantly, analyses showed that these data were not missing completely at random, and the results should be interpreted with this limitation in mind. Third, the data analyzed for this study did not contain all three measures (adiposity, inflammatory activity, and working memory) at three or more time points; therefore, we were unable to analyze the data in more sophisticated longitudinal models, such as cross-lagged panel or latent growth models. Relatedly, because working memory was only measured at one timepoint, our data cannot address change in working memory. Although adolescents show mean-level and rank-order changes in working memory over time (e.g., Boelema et al., 2014; Tamnes et al., 2013; Lensing and Elsner, 2018), it is not appropriate to infer that our sample showed changes in working memory ability over the assessments in this study. Although prior experimental work has found that reducing adiposity enhances executive functions such as working memory (Siervo et al., 2011; Vantieghem et al., 2018), our data do not permit inferences that adiposity predicts change in working memory. Nonetheless, our data permit the inference that individual differences in working memory relate to changes in both adiposity and CRP, as working memory predicted levels of these variables at a later timepoint when controlling for their values at an earlier timepoint (i.e., residualized changes). Fourth, we were not able to assess how other cognitive functions potentially important to obesity, such as inhibitory control, related to adiposity and inflammatory activity over time. Fifth, many potential confounds—such as nutritional supplements and diet—were not controlled. Although covarying hospitalizations from ages 9 to 13 and other potential confounds did not alter our results, it is possible that these results would differ if additional potential confounds were controlled. Finally, a major shortcoming of this study is that CRP and adiposity were assessed at the same points in time, which precludes inference of temporal precedence between these variables and entails that the mediation paths between adiposity and CRP were not longitudinal. Therefore, although longitudinal inferences can be made in how working memory relates to adiposity or CRP over time, our mediation analyses are not wholly longitudinal. Strengths of this study, in contrast, include its longitudinal design, large sample size, use of the gold standard DEXA to quantify adiposity, and the important theoretical contribution made by this study through its examination of bidirectional links between adiposity, inflammatory activity, and working memory over time.

In sum, we examined links between adiposity, inflammatory activity, and working memory across time in a large sample of 8536 children from the UK followed through adolescence. We hypothesized and found that there would be two pathways linking adiposity, inflammatory activity, and working memory over time. In the first of these pathways, adiposity predicted poorer working memory over time via their shared links to inflammatory activity. The second pathway linked poorer working memory to subsequent inflammatory activity in part through adiposity. These findings therefore suggest a scenario by which adiposity, inflammatory activity, and working memory may influence each other within a positive feedback loop over time, with adiposity leading to poorer working memory via inflammatory activity, and poorer working memory leading to greater adiposity and inflammatory activity over time. The rise of excess weight is a major concern for public health, and our results suggest that inflammatory activity and working memory may play an important role in its development and maintenance.

## Author note

We are extremely grateful to all the families who took part in this study, the midwives for their help in recruiting them, and the whole ALSPAC team, which includes interviewers, computer and laboratory technicians, clerical workers, research scientists, volunteers, managers, receptionists and nurses. The UK 10.13039/501100000265Medical Research Council and 10.13039/100004440Wellcome (Grant ref: 217065/Z/19/Z) and the 10.13039/501100000883University of Bristol provide core support for ALSPAC. A comprehensive list of grants funding is available on the ALSPAC website (http://www.bristol.ac.uk/alspac/external/documents/grant-acknowledgements.pdf). This research was specifically funded by MRC Grant G0401540 73080 to the ALSPAC team, an NSFIBSS 1327768 (PI: Page) and UC Davis Interdisciplinary Frontiers in the Humanities and Arts (IFHA) to P. D. Hastings, and R01 HD 093898 (PI: Bitler) to C. E. Hostinar. This publication is the work of the authors (Shields, Deer, Hastings, and Hostinar), who will serve as guarantors for the contents of this paper.

Grant S. Shields is now in the Department of Psychological Science at the University of Arkansas.

## Footnotes

1 In particular, a series of logistic regressions found that CRP missingness at age 9 was related to fat mass at age 9 (*p* ​< ​.001); working memory missingness at 10 was predicted by CRP at 9 (*p* ​< ​.001); fat mass missingness at 15 was predicted by fat mass at 9, CRP at 9, hospitalizations since 9, puberty at 15, and working memory at 10 (*p*s ​< ​.020); and CRP missingness at 15 was predicted by puberty at 15 and CRP at 15 (*p*s ​< ​.002).

2 This model and the subsequent model including covariates did not include paths from adiposity at age 9 to CRP at age 15.5 or from CRP at age 9 to adiposity at age 15.5 because we were interested in examining the two pathways for which we had *a priori* predictions. Including these aforementioned paths in the models did not alter any of the results (e.g., with and without including covariates, all total and indirect path *p*s ​≤ ​.028): All significant paths reported remained significant, and all nonsignificant paths reported remained nonsignificant.

3 Because CRP values greater than 10 ​mg/L can indicate current infection, chronic disease, or injury (Mac Giollabhui et al., 2020; O’Connor et al., 2020), we also ran the analyses excluding CRP values greater than 10 ​mg/L (*n* ​= ​60 ​at age 9, *n* ​= ​61 ​at age 15.5, *n* ​= ​3 ​at both assessments). With values excluded, both mediations remained significant. In particular, in Pathway 1, the path from adiposity at age 9 to CRP at age 9 was significant, β ​= ​0.459, *p* ​< ​.001, as was the path from CRP at age 9 to working memory at age 10, β ​= ​−0.045, *p* ​= ​.009; most importantly, the indirect association between adiposity at age 9 and working memory at age 10 through CRP at age 9 was significant, β ​= ​−0.021, *p* ​= ​.009. Similarly, in Pathway 2, the path from working memory at age 10 to adiposity at age 15.5 was significant, β ​= ​−0.024, *p* ​= ​.013, as was the path from adiposity at age 15.5 to CRP at age 15.5, β ​= ​0.209, *p* ​< ​.001; most importantly, the indirect association between working memory at age 10 and CRP at age 15.5 through adiposity at age 15.5 was significant, β ​= ​−0.005, *p* ​= ​.015.
